# Sustainability of the whole-community project '10,000 Steps': a longitudinal study

**DOI:** 10.1186/1471-2458-12-155

**Published:** 2012-03-05

**Authors:** Ragnar Van Acker, Ilse De Bourdeaudhuij, Katrien De Cocker, Lisa M Klesges, Annick Willem, Greet Cardon

**Affiliations:** 1 Department of Movement and Sport Sciences, Ghent University, Watersportlaan 2, 9000 Gent, Belgium; 2 School of Public Health, University of Memphis, 236A Robison Hall, Memphis, USA

## Abstract

**Background:**

In the dissemination and implementation literature, there is a dearth of information on the sustainability of community-wide physical activity (PA) programs in general and of the '10,000 Steps' project in particular. This paper reports a longitudinal evaluation of organizational and individual sustainability indicators of '10,000 Steps'.

**Methods:**

Among project adopters, department heads of 24 public services were surveyed 1.5 years after initially reported project implementation to assess continuation, institutionalization, sustained implementation of intervention components, and adaptations. Barriers and facilitators of project sustainability were explored. Citizens (*n *= 483) living near the adopting organizations were interviewed to measure maintenance of PA differences between citizens aware and unaware of '10,000 Steps'. Independent-samples *t*, Mann-Whitney *U*, and chi-square tests were used to compare organizations for representativeness and individual PA differences.

**Results:**

Of all organizations, 50% continued '10,000 Steps' (mostly in cycles) and continuation was independent of organizational characteristics. Level of intervention institutionalization was low to moderate on evaluations of routinization and moderate for project saturation. The global implementation score (58%) remained stable and three of nine project components were continued by less than half of organizations (posters, street signs and variants, personalized contact). Considerable independent adaptations of the project were reported (e.g. campaign image). Citizens aware of '10,000 Steps' remained more active during leisure time than those unaware (227 ± 235 and 176 ± 198 min/week, respectively; *t *= -2.6; p < .05), and reported more household-related (464 ± 397 and 389 ± 346 min/week, respectively; *t *= -2.2; p < .05) and moderate-intensity-PA (664 ± 424 and 586 ± 408 min/week, respectively; *t *= -2.0; p < .05). Facilitators of project sustainability included an organizational leader supporting the project, availability of funding or external support, and ready-for-use materials with ample room for adaptation. Barriers included insufficient synchronization between regional and community policy levels and preference for other PA projects.

**Conclusions:**

'10,000 Steps' could remain sustainable but design, organizational, and contextual barriers need consideration. Sustainability of '10,000 Steps' in organizations can occur in cycles rather than in ongoing projects. Future research should compare sustainability other whole-community PA projects with '10,000 Steps' to contrast sustainability of alternative models of whole-community PA projects. This would allow optimization of project elements and methods to support decisions of choice for practitioners.

## Background

Prevention programs represent an important strategy for improving public health, but their effectiveness rests on the extent to which they are evidence-based and can be widely disseminated and implemented [1,2]. The extent to which an evidence-based health program can deliver its intended benefits over an extended period of time after external (i.e. financial, managerial, or technical) support is terminated has recently been described as 'sustainability', and dimensions of sustainability are situated at individual, organizational and community levels [3]. Dissemination and implementation specific to PA programs have been considered a staged process with sustainability representing a later stage in these programs' life cycle but dependent on success at earlier stages [4,5]. An alternative model on the temporal aspects of sustainability argues that program implementation and sustainability occur concomitantly rather than in stages [6]. Sustainability is particularly relevant for public health practitioners and policy makers because investments in public PA programs need to be efficient, and lack of sustainability could lead to an investment loss [5,6].

Research into sustainability is considered a key criteria for determining the generalizability or external validity of PA programs [7,8]. However, recent systematic reviews of external validity information in studies of community-wide PA interventions concluded that most studies lacked reports of sustainability [9,10]. Lack of reported sustainability information at both the individual and organisational levels in community-wide studies is problematic because it limits the estimation of the long-term impact of such interventions [9]. Additionally, the extent of sustainability is also underreported, including information about which specific intervention components were retained, which were discontinued, and why [11].

The extent to which intervention components are modified or adapted by organizations to suit community contexts is also considered very relevant for sustainability research [5,12]. From a dynamic systems perspective, organizations operate in specific contexts where they are engaged in interactions with health programs and the community, and these interactions may adapt to new conditions [12,13]. This dynamic systems perspective implies that organizations would shape PA programs to their specific (community) context or to changing circumstances [13]. Documenting these adaptations can facilitate the dissemination of best practices and therefore promote sustainability [14].

**Figure 1:**
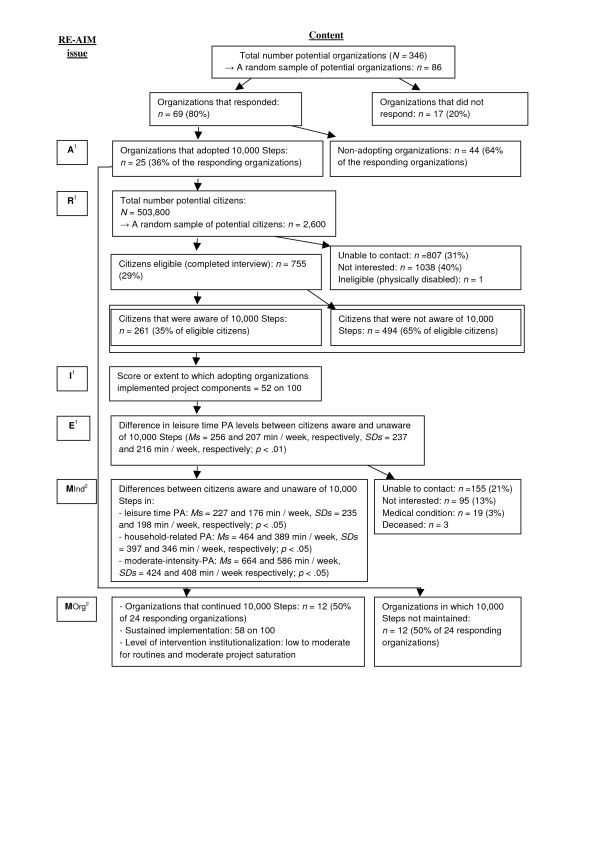
**Flow chart for the different dimensions of the RE-AIM framework applied to the'10,000 Steps' project**. R = Reach, E = effectiveness, A = adoption, I = implementation, M_ind _= maintenance at individual level, M_Org _= maintenance at organizational level. ^1 ^Results of the initial evaluation with the RE-AIM framework [15]. ^2 ^Results of the current sustainability study.

In response to the dearth of information in dissemination and implementation literature about the sustainability of community-wide PA programs in general and of '10,000 Steps' in particular, the Government of Flanders (Dutch speaking part of Belgium) provided funding to study the sustainability of '10,000 Steps' in the Flemish region. The '10,000 Steps' project in Flanders is a whole-community project including multiple community-based strategies to promote PA in the adult population [15]. Its intervention components constitute a socio-ecological approach [16], including personal (e.g. pedometer sale and loan, dissemination of information), organizational (e.g. organization of community events), community (e.g. street signs, local media campaign) and policy (e.g. partnerships with other stakeholders) components. Efforts to disseminate '10,000 Steps' in Flanders were initiated in the last quarter of 2007. Dissemination strategies focused primarily on the use of media and peer networks of public health and sports services based on the principles of Roger's Diffusion of Innovations [15,17]. The dissemination and implementation of '10,000 Steps' was studied in 2009, after which funding for project dissemination ended [15]. Using the RE-AIM framework (reach, effectiveness, adoption, implementation, maintenance) [18] for evaluation, a project adoption rate of 36% and a global implementation score of 52% was measured in professional organizations at that time, while effectiveness measures showed that citizens aware of '10,000 Steps' had significantly more leisure time PA than citizens unaware of it. Figure 1 shows that the initial evaluation with the RE-AIM framework provided final results for 4 out of 5 dimensions including reach, effectiveness, adoption and implementation. Further details of the initial evaluation with the RE-AIM framework are provided elsewhere [15]. However, measures of sustainability or the 'maintenance' dimension in the RE-AIM framework were premature at the time of data collection and follow-up measurements for that particular dimension were recommended [15]. Such follow-up measurements could expand the limited knowledge on the potential of whole-community projects like '10,000 steps' to be continued and institutionalized in organisations after dissemination, and to sustain behavioural effects. Therefore, the aim of the current study was to evaluate sustainability-indicators of '10,000 Steps' at organizational and individual levels, two and a half years after project dissemination and more than 1 year after the prior dissemination and implementation study.

## Methods

### Sample and data collection

Participants for this sustainability study were situated at organizational (i.e. organizations) and individual levels (i.e. citizens). In the prior dissemination and implementation study of '10,000 Steps', 25 public health and sports services department heads reported the extent of project adoption before the first quarter of 2009 [15]. The same group was recontacted 17 months later by email for follow-up responses. After a pre-notification email the organizations received a web link to a code-protected online survey. Three reminder emails within a 5 week period were sent to non-responding organizations. A total of 24 of 25 professional organizations participated in the online survey (96% response rate). Their public service organizations served an approximate total of 531,687 adult citizens.

On the individual level, the 755 respondents of the dissemination and implementation study of '10,000 Steps' [15] were contacted at 14 months follow-up and invited to complete a telephone interview. These respondents were adult citizens living in the work area of the participating professional organizations that had adopted '10,000 Steps' as a whole-community project before the first quarter of 2009. Three attempts were made before recording individuals as "not contactable". A total of 502 citizens completed telephone interviews (66% response rate), 155 were not contactable (21%), 95 were not interested (13%) and 3 were deceased. After excluding ineligible respondents (*n *= 19, due to events potentially influencing PA levels since the previous time of data collection, such as heart surgery) the final sample consisted of 483 citizens.

All data were collected in the second quarter of 2010. Study protocols were approved by the Ethical Committee of the Ghent University.

### Measures

Sustainability measures of '10,000 Steps' in the follow-up organizational survey included 1) project continuation (including sustained implementation of project components and adaptation) and 2) institutionalization in organizations (see Additional file 1). Individual sustainability indicators were established through the telephone interviews and included maintenance of the project's initial effects on individuals' PA [3,13].

#### Project continuation

Project continuation was defined as the proportion of organizations that reported continuation of '10,000 Steps' project, in part or in whole, after the prior dissemination and implementation study (Did your organization continue the adoption of '10,000 Steps' the past year?) [9]. Reasons for continuing or declining continuation of the project were also questioned. Additionally, number of staff members, type of organization and working context were questioned to assess representativeness [7].

##### Sustained implementation of the nine project components

Sustained implementation was measured as the proportion of organizations that continued a specific project or intervention component of '10,000 Steps'. The project components, representing nine socio-ecological components for PA promotion, reflect the key intervention strategies applied in the pilot study in the city of Ghent and the dissemination and implementation study of '10,000 Steps' in the Flanders region [15,19]. These components include: 1) the sale or loan of pedometers in public places (Did your organization sell or loan pedometers during the implementation of '10,000 Steps'), 2) the use of the '10,000 Steps' website (Did your organization use the supportive website for '10,000 Steps', http://www.10000stappen.be?), 3) repeated dissemination of information using variants of flyers (Did your organization disseminate flyers of '10,000 Steps'?) and 4) posters in public places (Did your organization disseminate posters of '10,000 Steps'?), 5) wide-ranging personal contact with citizens (Did your organization contact citizens in a personalized manner (e.g. personalized letter, mail, or phone)?), 6) the organization of community events (Did your organization stage any community events to promote '10,000 Steps'?), 7) repeated use of the media (Did your organization conduct a media campaign to promote '10,000 Steps'?), 8) the repeated or permanent use of street signs or other strategically placed objects in the street scene (e.g. bill boards) to encourage PA (Did your organization put street signs, billboards, or other promotional materials of '10,000 Steps' in the street scene?) and 9) the initiation of partnerships with local authorities and other associations (Did your organization initiate any partnerships with municipal services, associations or societies to implement '10,000 Steps'?). The median implementation score (score on 100) across all nine components was taken as the global implementation score with methods described earlier [20]. Additional measures included project duration and investment as well as the number of domains of active living or PA domains that was targeted.

##### Adaptation

Project adaptation was evaluated as the extent to which organizations made changes to '10,000 Steps' from original content or added new project elements during implementation to fit their setting [3] (Did your organization modify any of the inquired project components or add any project elements to '10,000 Steps'? If yes, please specify the additions or modifications).

#### Institutionalization

Institutionalization or the extent to which'10,000 Steps' was integrated within the culture of organizations through policies and practice [3] was measured using the LoIn scales, developed by Goodman and colleagues [21] and further tested for reliability and validity by Barab and colleagues [22]. The LoIn scales measure 15 aspects of institutionalization related to project production (e.g. written project goals, evaluation), project maintenance (e.g. permanent staff), project support (e.g. permanent funding), and managerial aspects (e.g. supervision). Based on these 15 aspects mean scores for 'routines' and 'niche saturation' scores can be calculated. Routines represent the continued inclusion of the project in the organization's formal plans, allowing them to become routine. The routines score was interpreted as low (≤ 1), low to moderate (> 1 and ≤ 3), moderate to high (> 3 and ≤ 5), and high (> 5). Niche saturation reflects the maximum beneficial expansion of the project within the organization. The saturation score was interpreted as low (≤ 2), moderate (> 2 and ≤ 3), and high (> 3) [21,22].

#### Maintenance

To evaluate whether initial effects were sustained on the longer term [7], PA levels were compared between citizens aware of '10,000 Steps' (reached citizens) and those not aware (citizens not reached), as was done in the prior dissemination and implementation study [15]. Citizens' PA levels in a usual week were re-assessed using the telephone-administered long version of the International Physical Activity Questionnaire (IPAQ). This included PA at work, transport related PA, gardening and domestic activities, and leisure time PA. The IPAQ has been proven to be a reliable and valid instrument for assessing PA at the population level in Europe and in Flanders, Belgium [23,24]. Project awareness was assessed with the following question: Have you heard of '10,000 Steps'? (yes/no) (see Additional file 2).

### Data analysis

The proportion of organizations that continued any aspect of '10,000 Steps' as well as which specific components were sustained was calculated. Differences between organizations continuing and not continuing '10,000 Steps' were analyzed with Mann-Whitney *U *tests and chi-square tests. Descriptive statistics provided information about reasons for (not) continuing '10,000 Steps' (*project continuation*).

The global implementation score and the implementation scores of each project component were also calculated and converted to *z*-scores. Comparisons of global and separate project components were made between initial implementation proportions with the follow-up proportions (i.e. comparison of implementation scores over time) using Wilcoxon tests (*sustained implementation*). Descriptive statistics were used to analyze reasons for not implementing components (*sustained implementation*) and to obtain insights in any modifications or additions to the project (*adaptation*).

Chi-square tests assessed differences over time in the proportion of organisations targeting the different PA domains, as well as differences over time in project duration. Wilcoxon tests were used to analyze differences over time in project investment.

LoIn scales were first scored and summed, and mean scores of routines and niche saturation were calculated (*institutionalization*) [21,22].

On the individual level, total time for PA in the four PA domains and total time for walking, moderate PA and vigorous PA, all expressed in minutes/week, were computed http://www.ipaq.ki.se at follow-up. Baseline PA levels were compared between drop-outs and respondents using independent samples *t *tests. Drop-out representativeness was analyzed with independent samples *t *tests and chi-square tests by comparing both groups on demographic characteristics of gender, age, employment status and educational level. Differences in PA levels between citizens aware and not aware of '10,000 Steps' were analyzed with independent samples *t *tests. By subtracting mean PA levels of citizens in both groups (citizens aware and not aware) and dividing this score by the pooled standard deviation of PA levels effect sizes for PA were computed. Effect sizes (*d*) were interpreted as negligible (< 0.15), small (0.15 - 0.40), medium (0.40 - 0.75) or large (> 0.75) [25]. Citizens aware and not aware of '10,000 Steps' were also compared on demographic characteristics using independent samples *t *tests and chi-square tests.

All analyses were performed in SPSS 15.0 (SPSS, Inc. Chicago, IL) and the level of significance was set at p ≤ 0.05.

## Results

Figure 1 illustrates the combined results for the sustainability measures of '10,000 Steps' in the current study and for the initial evaluation with the RE-AIM framework. In this section results of the current sustainability study will be reported in more detail.

### Sample of organizations and citizens

Organizations at follow-up evaluation included 12 health insurance organizations (of 12 contacted and 26 at baseline), 6 local health promotion services (of 7 contacted and 18 at baseline), and 6 municipal sports services (of 6 contacted and 25 at baseline). Compared to the general population in Flanders, responding citizens (*n *= 483) had a smaller proportion of men (49.0% vs. 39.5%, respectively), a somewhat higher mean age (48.0 years vs. 51.0 years, respectively), a similar proportion of employed people (66.3% vs. 74.0%, respectively), and a higher proportion of persons with a higher education degree (25.0% vs. 41.9%, respectively). No significant differences between drop-outs and respondents in the total time for PA within the four domains of active living and total time for walking, moderate, and vigorous PA were seen. Compared to respondents, drop-outs also had a similar proportion of men (39.5% vs. 43.4%, respectively), a similar mean age (51.0 years vs. 50.0 years, respectively), a similar proportion of employed people (74.2% vs. 74.0%, respectively), and a similar proportion of persons with a higher education degree (41.9% vs. 35.7%, respectively).

### Project continuation

Of all organizations, 50% (12 of 24) reported continuation of '10,000 Steps' almost one and a half year after the prior dissemination and implementation study. Comparisons between organizations continuing and not continuing '10,000 Steps' revealed no significant differences in the mean number of staff members [*Ms = *4.3 vs. 3.9, respectively, *SDs *= 3.3 vs. 2.6, respectively; Mann-Whitney *U*-test *z *= 0.60, *p *= 0.86], type of organization [83.3% vs. 66.7% with a health policy focus, respectively; χ^2^(1) = 0.89, *p *= 0.35] and working context [16.7% vs. 16.7% only rural, respectively; χ^2^(1) = 0.00, *p *= 1.00]. Five out of twelve organizations reported they continued'10,000 Steps' as an ongoing project or one that lasted more than 1 year (42%; *n *= 5). All other organizations reported periodically repeated projects (cycles) with a project duration of 3 to 12 months (42%; *n *= 5) or less than 3 months (16%; *n *= 2).

Organizations continuing '10,000 Steps' and those that had the intention to do so in the future gave the following main reasons for continuation: 'assignment given by the organization's superior' (46%), 'external funding or support from other stakeholders' (39%), '10,000 Steps' is a ready-for-use product' (23%), and 'potential of '10,000 Steps' to be adapted for specific groups (e.g. low SES)' (23%). Organizations not continuing '10,000 Steps' also listed reasons for not having the intention or having doubts to continue '10.000 Steps' including: 'not having thought about it yet in a concrete manner' (33%), 'preference for another PA project' (33%), 'no priority or not suitable' (11%) and 'insufficiently planned resources' (11%).

### Sustained implementation

Three of nine separate components of '10,000 Steps' were continued by less than half of organizations (Table 1). These components included the use of posters in public places, the use of street signs or other strategically placed objects in the street scene, and wide-ranging personal contact with citizens.

**Table 1:** Proportion of organizations that continued a specific project component of 10,000 Steps

Project component	Proportion
Dissemination of information using variants of flyers	100% (10 of 10)
Use of the media to promote '10,000 Steps'	88% (7 of 8)
Sale or loan of pedometers	83% (10 of 12)
Organization of community events	83% (5 of 6)
Initiation of partnerships with local authorities and other associations	75% (6 of 8)
Use of the '10,000 Steps' website	57% (4 of 7)
Use of posters in public places	33% (2 of 6)
Use of street signs or other strategically placed objects in the street scene	25% (1 of 4)
Wide-ranging personal contact with citizens	0% (0 of 3)

Compared to the prior dissemination and implementation study [15] the global implementation score of organizations continuing '10,000 Steps' remained stable in this sustainability study (a median 58 of 100) and no significant differences were found for implementation scores on the separate components (see Table 2 for corresponding *z*-scores). Most frequently reported reasons for not having implemented components across both studies are indicated in Table 3.

**Table 2:** Implementation scores of organizations continuing 10,000 Steps: comparison between prior study and sustainability study

	Prior dissemination and implementation study		Present sustainability study		
		
Project component	Implementation score on 100 (mean ± SD)	z-score	Implementation score on 100 (mean ± SD)	z-score	p-value
Organization of community events	85.7 ± 37.8	1.12	85.7 ± 37.8	1.28	1.00
Sale or loan of pedometers	100.0 ± 0.0	1.67	83.3 ± 38.9	1.18	0.16
Dissemination of information using variants of flyers	62.5 ± 37.7	0.23	66.7 ± 24.7	0.54	0.74
Initiation of partnerships with local authorities and other associations	54.2 ± 45.0	-0.09	62.5 ± 48.3	0.38	0.48
Use of the media to promote '10.000 Steps'	58.3 ± 46.9	0.07	58.3 ± 41.7	0.22	1.00
Use of posters in public places	45.8 ± 49.8	-0.41	48.6 ± 42.9	-0.16	0.87
Use of the '10.000 Steps' website	58.3 ± 51.5	0.07	38.9 ± 47.3	-0.54	0.28
Use of street signs or other strategically placed objects in the street scene	29.2 ± 45.0	-1.05	16.7 ± 32.6	-1.4	0.45
Wide-ranging personal contact with citizens	14.3 ± 19.7	-1.62	14.3 ± 37.8	-1.49	0.71

*Global implementation score (median)*	*58.3*		*58.3*		

**Table 3:** Components with lowest implementation scores across (the prior and sustainability) studies and reasons for non-implementation

Project component	Reported reasons for non-implementation (% of organizations)
Use of posters in public places	Posters not available on time (production and planning problem) (50%)
	No time (25%)
	Still considering implementation (25%)
Use of street signs or other strategically placed objects in the street scene	Not relevant to our core business (44%)
	Too expensive (33%)
	No time (11%)
Wide-ranging personal contact with citizens	Not relevant to our core business (50%)
	Other health-related priorities (e.g. mental health) (17%)
	No time (17%)

When considering the targeted PA domains, similar proportions of organizations targeted leisure time in this sustainability study compared to the prior dissemination and implementation study [100.0% vs. 91.7%, respectively; χ^2^(1) = 1.04, *p *= 0.31]. Significantly fewer organizations marked work-related activities in this sustainability study compared to the prior dissemination and implementation study [8.3% vs. 66.7%, respectively; χ^2^(1) = 8.71, *p *< 0.01] as well as household activities [33.3% vs. 83.3%, respectively; χ^2^(1) = 6.17, *p *< 0.05], and there was a trend towards targeting active transport less frequently than in the prior dissemination and implementation study [58.3% vs. 91.7%, respectively; χ^2^(1) = 3.56, *p *= 0.06].

No significant differences between this sustainability study and the prior dissemination and implementation study were found in number of working days organizations devoted on '10,000 Steps' [Ms = 38.5 days vs. 53.4 days, respectively, *SDs *= 40.1 vs. 83.6, respectively; Wilcoxon *z *= -1.37, *p *= 0.17] or the estimated financial investment for project implementation [Ms = 0.016 euros vs. 0.026 euros per citizen, respectively, *SDs *= 0.018 vs. 0.021, respectively; Wilcoxon *z *= -1.60, *p *= 0.11]. The proportion of organizations reporting a project duration of more than 6 months was identical in this sustainability study and the prior dissemination and implementation study [41.7% vs. 41.7%, respectively; χ^2^(1) = 0.00, *p *= 1.00].

### Adaptation

Specific project adaptations by organizations included replacing or supplementing posters in public places (17%) with exhibition banners and foot stickers (high visible foot-shaped stickers on floors of public places). The campaign image of all organizations continuing '10,000 Steps' was different from the campaign image promoted during the prior dissemination study. Fifty-eight percent of organizations used their own campaign image (or an image developed by stakeholders of partner organizations), 58% (also) used the campaign image of a new '10,000 Steps' project funded by the Flemish government, and 8% used no image.

When considering the targeted populations, a minority of organizations changed the project focus from a specific target group (e.g. the elderly or personnel only) to a whole-community approach (17%) or vice versa (8%).

### Institutionalization

Institutionalization scales [18] were low-to-moderate for routines or the way '10,000 Steps' was routinized (M = 1.31, *SD *= 0.56) and moderate for the degree of project saturation in organizations (M = 2.03, *SD *= 0.71).

### Maintenance

The group of citizens aware of '10,000 Steps' reported significantly higher leisure time and household PA levels than those not aware (Table 4). In the different categories of PA intensity, citizens aware of '10,000 Steps' also reported significantly more minutes of moderate PA per week than those not aware. Citizens that were aware of '10,000 Steps' had a smaller proportion of men [47.2% vs. 52.8%, respectively; χ^2^(1) = 6.89, p < 0.01], a similar mean age [*Ms *= 50.4 years vs. 51.4 years, respectively, *SDs *= 16.1 vs. 13.7, respectively; *t*(325) = -0.69, *p *= 0.49], a similar proportion of employed people [71.9% vs. 75.5%, respectively; χ^2^(1) = 0.59, *p *= 0.44], and a similar proportion of persons with a higher education degree [41.2% vs. 42.2%, respectively; χ^2^(1) = 72.45, *p *= 0.83] compared to citizens unaware of the project.

**Table 4:** Mean physical activity (PA) levels for respondents who were aware and unaware of 10.000 Steps

	Group aware of '10,000 Steps' n = 305 (63%)	Group unaware of '10,000 Steps' n = 178 (37%)	df	t	d
**Transport-related PA (min/week)**	142 ± 187	115 ± 175	481	-1.5	0.15
**Leisure time PA (min/week)**	227 ± 235	176 ± 198	421	-2.6*	0.23
**Household PA(min/week)**	464 ± 397	389 ± 346	411	-2.2*	0.20
**Work-related PA (min/week)**	221 ± 382	255 ± 427	481	0.9	0.08
**Walking (min/week)**	258 ± 290	231 ± 263	481	-1.0	0.10
**Moderate PA (min/week)**	664 ± 424	586 ± 408	481	-2.0*	0.19
**Vigorous PA (min/week)**	66 ± 141	86 ± 168	481	1.4	0.13

*p < 0.05

*d*: effect size

## Discussion

This longitudinal study measured the sustainability of the whole-community project '10,000 Steps' in extension of our previous research on this project's dissemination and implementation process in Flanders [15]. It contributes additional findings to the limited research on sustainability of community-wide PA programs available in the literature [9,10].

Levels of project continuation among organizations were 50% which was 10% lower than the 60% benchmark reported by a review on empirical sustainability studies of health-related programs [11]. However, this comparison should be interpreted with caution because the types of programs reviewed varied considerably and may not be directly comparable to '10,000 Steps'. It has been hypothesized that complex interventions, such as whole-community projects, that call for changes in different socio-ecological levels of dynamic systems (i.e. communities) are more difficult to sustain [12]. Further research on the sustainability of other whole-community projects promoting PA could explore this hypothesis.

Conceptual frameworks argue that sustainability is determined by a combination of factors related to project design, organizational setting, and the broader context [5,26,27]. Findings of the present study support these frameworks and suggest several factors particularly important for increasing the sustainability of whole-community projects like '10,000 Steps'. Reported reasons for project continuation indicate the importance of offering ready-for-use and adaptable interventions to public service organisations including project manuals, materials and websites (project design factors), targeting the organization's superior as a project champion (organizational setting), and providing funding or external support from other stakeholders (broader context). A major reported barrier to project continuation was the preference to switch to another PA project. Next to a lack of project compatibility with the perceived needs of reporting organizations, this finding suggests a need for organizations to have a choice in their decision-making about project implementation. Dearing and colleagues [28] argue that increasing project choice is a key principle for disseminating proven approaches to PA promotion. A logical recommendation would be to combine sustainability strategies of '10,000 Steps' with strategies that cluster alternative evidence-based programs [28]. The web portal Cancer Control PLANET (Plan, Link, Act, and Network with Evidence-based Tools) is a practical example of this principle [29]. Considering external validity indicators in the present study, these were promising, with project continuation rates found to be independent of staff size, type of organization and working context. The global implementation score (58%) remained stable and the majority of project components were continued by more than half of organizations. It is encouraging that partnerships in particular were continued by 75% of organizations as partnerships with other stakeholders are argued to have a positive influence on long-term sustainability of projects [11,14]. Three project components (personal contact, street signs and variants, and posters) were continued by less than half of organizations. These same components also had low implementation rates with negative *z*-scores in both the current study and the prior dissemination and implementation study. Personal contact with citizens seemed infeasible for a substantial portion of organizations, which is consistent with findings of a smaller scale dissemination study of an evidence-based PA promotion program for the elderly in community organizations [30]. Mixed reasons, including costs, were listed for non-implementation of street signs or variants, implying the need for alternatives. After similar reasons had been reported in the prior dissemination and implementation study, the Flemish Government initiated an intermediate support structure for '10,000 Steps' on the regional level. This intermediate structure consisted of linkage agents (health promotion services) who developed more workable alternatives such as street banners and flags. However, because planning time for both the intermediate structure and alternative materials was underestimated, availability of these materials was delayed by 1 year. The potential of street banners and flags could therefore not be fully assessed. Production delays of other project materials on the regional level were directly reflected in listed reasons for not continuing the use of posters. Several organizations listed dependency of poster production on the regional level which forced them to continue '10,000 Steps' without posters. This underlines the importance of synchronizing project strategies and change across different levels of a social system to optimize sustainability, as also suggested by Schensul [12]. When considering the targeted PA domains, it was clear that leisure time was the organizations' preferred context to continue the promotion of '10,000 Steps', followed by transport, household, and work.

Several organizations changed the project focus of '10,000 Steps' and switched between a whole-community approach and a specific target group (e.g. the elderly). This emphasizes that sustainability is a dynamic process and strategies for achieving it ought to adapt to changing contexts and priorities [5]. In order to facilitate this dynamic process, project manuals should include extensive guidelines for project adaptations that don't jeopardize project fidelity. This includes specific manuals for projects targeting the elderly only, or projects in workplaces. Furthermore, many organizations continuing '10,000 Steps' changed the original campaign image into one that reflected their regional or local identity (e.g. colours, partner logos, and multiple images). As argued by Dearing and colleagues [28], it is important that projects provide organizations more opportunities to raise their profile and to feel project ownership as a strategy to encourage project sustainability. Furthermore, organizations reported alternatives for less sustainable project components, such as exhibition banners or foot stickers in public places instead of regular posters. This kind of information is useful for formulating guidelines on how to reinforce or replace less sustainable components with alternatives that retain the original function of promoting awareness and knowledge about PA. Future sustainability studies of community-wide PA programs could facilitate the spread of best practices for various settings by documenting adaptations and whether these adaptations retain essential behaviour change elements [7,14].

Institutionalization levels for routines and project saturation indicate that '10,000 Steps' became moderately integrated within the culture of organizations through policies and practice. However, two separate aspects of institutionalization were poorly integrated. Few permanent staff members contributed to project implementation, which could jeopardize sustainability in case of staff turnover. In addition, very few organizations reported formal project evaluation. As evaluation was not explicitly recommended in the initial '10,000 Steps' project findings suggest it is necessary to do so in the future. Wilson and Kurz [31] argue that lack of evaluation is a significant threat to institutionalization. Without evaluation, the project's potential benefits cannot be monitored, which may reduce relevancy of the project within the organization. In order to facilitate project evaluation and sustainability a combination of measures is recommended. First, Wilson and Kurz [31] recommend providing organizations with training and practical methods for continuous evaluation and quality improvement, such as the "Plan-Do-Study-Act" framework [32]. Second, this study also supports Brug and colleagues [33] who argue the importance of better planning, considerable more funding opportunities for evaluation, and the formation of collaborative centers between public health services and academic experts.

Seven out of twelve organizations reported to have sustained '10,000 Steps' by implementing periodically repeated projects rather than ongoing projects. This implies that project sustainability in organizations can also manifest itself in repeated time cycles rather than on a continuous basis only. This alternative approach to the time dimension of sustainability seems more logical and appropriate for projects like '10,000 Steps' which aim to increase citizens' awareness about PA. For example, project components such as street banners may be disregarded by citizens after years of permanent allocation. Such components may grasp interest of citizens in a more sustainable manner when implemented repeatedly in cycles and as part of a comprehensive intervention [34]. As discussed previously, it would be recommendable to offer project choice to organizations in public health. This would allow organizations to alternate cycles of '10,000 Steps' with cycles of other evidence-based PA programs. This also implies that projects like '10,000 Steps' would need to innovate regularly (e.g. by anticipating and facilitating adaptations) to sustain high levels of interest in both adopters and citizens. The optimal duration or schedule for project cycles of '10,000 Steps' is probably different for each community context because sustainability is a dynamic process. However, based on the present study it seemed that periodically repeated projects can be feasible when they have a maximum duration of 1 year and are alternated with varying periods of interruption.

In the prior dissemination and implementation study, significant differences in domain-specific self-reported levels of PA were only found for leisure-time PA, with citizens aware of '10,000 Steps' found to have higher levels than those not aware [15]. No differences were found for intensity-specific self-reported levels of PA at that time. The same comparative analyses for the current sustainability study confirmed maintenance of significantly more leisure time PA in citizens aware of '10,000 Steps'. In addition, in the current study citizens aware of '10,000 Steps' also reported significantly more household-related and moderate intensity PA. Project materials of '10,000 Steps' promoted all PA domains and also the public health guideline of 30 min of moderate to vigorous intensity PA per day, which may have contributed to additional time-delayed effects in favor of citizens aware of '10,000 Steps'. Furthermore, results suggest (although not significant) that citizens that were unaware of '10,000 Steps' had more work-related PA and vigorous intensity PA. The latter finding could indicate higher fitness levels in these citizens. However the testing of fitness levels was beyond the scope of the current study.

Limitations of this study include the use of self-report data and reliance on only one respondent per organization. With more resources these approaches could become more valid by adding objective types of data collection (e.g. on-site observations) and by contacting multiple respondents per organization [11]. While the sample of organizations was relatively small, which may limit external validity of results, the sample itself appears to be generalizable. In the prior study of '10,000 Steps' [15] external validity analyses revealed no significant differences between these same organizations and the larger group (*n *= 44) of non-adopting organizations on organizational characteristics (e.g., size, type, and context). Moreover, despite their small number, organizations in the current study served substantial populations and could have wide-scale impact on public health. Finally, this study did not address the issue of capacity building and community level changes, which is considered a third indicator of sustainability [3]. For example, we did not explore if a capacity-building strategy for local staff members influenced the sustainability of '10,000 Steps' or the development of other PA programs. Green [35] argued that rather than program continuation and institutionalization the most appropriate goal of sustainability may be that practitioners learn new skills and new program approaches.

Strengths of this study include its contribution to a small available literature on the sustainability of community-wide physical activity (PA) programs in general and of '10,000 Steps' in particular. Sustained implementation of project components and adaptations were also assessed. This provided more insights in less sustainable components and its contextual barriers, which may also be relevant for other sustainability research. Reported adaptations provide additional information to potentially improve project sustainability in various and changing contexts.

## Conclusions

This study shows that projects like '10,000 Steps' could remain sustainable and that project sustainability in organizations can also manifest itself in periodically repeated projects (cycles) rather than ongoing projects (continuous). However, several important barriers need consideration. Limited project evaluation and permanent staff appointed to project implementation may jeopardize institutionalization, while insufficient planning time to optimize synchronization between regional and community policy levels may be harmful for sustained implementation of project components. To make whole-community PA projects like '10,000 Steps' more sustainable several strategies are proposed. These include obtaining support of program champions with upper-level managerial authority and 'packaging' projects as ready-for-use products, with ample space and guidelines for adaptation to various and changing contexts and priorities. Furthermore, maintaining funding that also meets practical needs for project evaluation (e.g. training and academic collaborative centres) and a regional (intermediate) support structure may be beneficial. Future dissemination and implementation research should examine the sustainability of other whole-community PA projects to compare with the present study. It would also be interesting to compare alternative dissemination and implementation approaches in which institutionalization efforts are concomitant with implementation [6]. This allows finding alternative whole-community PA projects, which is necessary to optimize project choice for practitioners. Additionally, the current study could be extended by including sustainability at the community level. Researching sustainability of whole-community approaches to PA promotion is worth the investment because they have potential for developing the social and cultural change required for sustained improvements in population PA [36].

## Competing interests

The authors declare that they have no competing interests.

## Authors' contributions

RVA designed the study, collected and analyzed the data, and drafted the manuscript. IDB participated in the design of the study and helped to draft the manuscript. KDC participated in the design, helped to interpret data and draft the manuscript. LK and AW helped to interpret data and draft the manuscript. GC participated in the design helped to interpret data and draft the manuscript. All authors read and approved the final manuscript.

## Pre-publication history

The pre-publication history for this paper can be accessed here:

http://www.biomedcentral.com/1471-2458/12/155/prepub

## Supplementary Material

Additional file 1 **Organizational survey**. Survey to assess organizational project continuation, sustained implementation, adaptation and institutionalization of '10,000 Steps'. Click here for file

Additional file 2 **Questionnaire for citizens**. Description: Questionnaire to assess individual project awareness and PA levels (including IPAQ). Click here for file
