# Supplementary material for: Sustainability of the whole-community project '10,000 Steps': a longitudinal study
Source: BMC Public Health. 2012 Mar 5;12:155. doi: 10.1186/1471-2458-12-155 (PMC3312865; doi:10.1186/1471-2458-12-155)
Supplement: Additional file 1 — Organizational survey. Survey to assess organizational project continuation, sustained implementation, adaptation and institutionalization of '10,000 Steps'. [file 1471-2458-12-155-S1.PDF]

UNIVERSITEIT GENT

Faculteit Geneeskunde en Gezondheidswetenschappen
Vakgroep Bewegings- en Sportwetenschappen
Watersportlaan 2
9000 Gent

Steunpunt beleidsrelevant onderzoek 2007-2011 cultuur, jeugd en sport

We stellen het ten zeerste op prijs dat u bereid bent om te helpen bij de implementatiestudie van "10 000 Stappen".

Uw antwoorden op de vragen in deze vragenlijst zijn zeer belangrijk voor de Vlaamse overheid en uw organisatie. Zij zullen bijdragen tot inzichten en aanbevelingen om projecten zoals "10 000 Stappen" beter af te stemmen op uw dienst.

Het afwerken van deze online vragenlijst zal +- 10 tot 35 minuten in beslag nemen.

Vergeet niet:

- wij willen weten wat u denkt
- er zijn geen goede of slechte antwoorden
- uw antwoorden zullen strikt vertrouwelijk behandeld worden

Bedankt voor uw deelname aan de implementatiestudie van "10 000 Stappen"!

Vriendelijke groeten,

Mast. en Lic. Ragnar Van Acker

Prof. Ilse De Bourdeaudhuij

Prof. Greet Cardon

DEEL 1 – TOEPASSING 10.000 STAPPEN

ALGEMENE INFORMATIE

1. Bij welke organisatie of dienst werkt u? (*kruis **1** bolletje aan*)

- O Gemeentelijke/stedelijke (sport)dienst
- O Ziekenfonds
- O sportdienst
- O Ander: ...

2. In welk type gebied is uw dienst actief? (*kruis **1** bolletje aan*)

- O In zowel stedelijk als landelijk gebied
- O Enkel in stedelijk gebied
- O Enkel in landelijk gebied

3. Hoeveel vaste werknemers telt uw dienst? ………… (vul in aub)

4. Is uw dienst op de hoogte van het project ‘10.000 Stappen’?

- O Neen
- O Ja ***(→ga naar vraag 5)***

Indien u zonet ‘Neen’ heeft geantwoord op deze vraag 4, <u>eindigt de vragenlijst hier</u>. Dank voor uw medewerking!

5. Sinds wanneer is uw dienst op de hoogte van ‘10.000 Stappen’? (*kruis **1** bolletje aan*)

- O sinds minder dan 6 maanden
- O sinds 6 maanden tot 1 jaar
- O sinds 1 tot 2 jaar
- O sinds 2 tot 3 jaar
- O meer dan 3 jaar

6. Langs welk kanaal is uw dienst geïnformeerd over ‘10.000 Stappen’? *(kruis **1** of **meerdere** bolletjes aan)*

O via collega’s uit een andere regio
O via Universiteit Gent of Steunpunt Cultuur, Jeugd en Sport
O via de Vlaamse coördinator van 10.000 stappen Vlaanderen
O via de Provincie
O via het ISB (congres of nieuwsbrief)
O Ander (*vul hieronder aan a.u.b.*):

………

7. Met welk informatiemiddel gebeurde dit? *(kruis **1** of **meerdere** bolletjes aan)*

O powerpointpresentatie rond 10.000 Stappen
O folder 10.000 Stappen
O website van 10.000 Stappen
O enkel mondelinge uitleg zonder bijkomend illustrerend materiaal
O Ander (*vul hieronder aan a.u.b.*):

……

8. Duid aan in welke mate u het eens bent met volgende uitspraken *(kruis **1** bolletje per regel aan)*

	**helemaal mee oneens**	**mee oneens**	**niet oneens/ niet eens**	**mee eens**	**helemaal mee eens**
De inhoud en beschikbare instrumenten van ’10.000 Stappen’ zijn goed gekend door uw dienst.	O	O	O	O	O
’10.000 Stappen’ betekent (kan) voor uw dienst een meerwaarde (betekenen) in vergelijking met andere, bestaande bewegingsprogramma’s/-projecten	O	O	O	O	O
’10.000 Stappen’ is complementair met de huidige doelstellingen en werking van uw dienst	O	O	O	O	O

	helemaal mee oneens	mee oneens	niet oneens/ niet eens	mee eens	helemaal mee eens
’10.000 Stappen’ komt tegemoet aan noden van uw dienst	O	O	O	O	O
’10.000 Stappen’ is geschikt om dit eerst op experimentele basis (kleine schaal) te organiseren en nadien eventueel uit te breiden	O	O	O	O	O
’10.000 Stappen’ is complex om te begrijpen en te organiseren	O	O	O	O	O
’10.000 Stappen’ levert (kan) visibiliteit en zichtbare resultaten op (opleveren)	O	O	O	O	O
’10.000 Stappen’ is een geschikt product om zich als dienst/organisatie te kunnen profileren	O	O	O	O	O

TOEPASSING - ALGEMEEN

9. Heeft uw sportdienst in de periode april 2009 tot april 2010 '10.000 Stappen' of een variant hiervan verder toegepast? (*kruis **1** bolletje aan*)

- O Neen, en we hebben ook geen intentie om dit in de toekomst te doen. ***(→ga nu naar vraag 11)***
- O Neen, en we weten nog niet of we dit zouden doen in de toekomst ***(→ga nu naar vraag 11)***
- O Neen, maar we hebben wel de intentie om dit in de toekomst te doen. **(*→ga nu naar vraag 12*)**
- O Ja, maar dit is reeds achter de rug
- O Ja, dit is nog gaande ***(→ga nu naar vraag 12)***

10. Hoe lang is het geleden dat uw '10.000 Stappen'-campagne of een variant hiervan is beëindigd? (*kruis **1** bolletje aan*)

- O tot 1 maand geleden ***(→ga nu naar vraag 12)***
- O 1 tot 3 maanden geleden ***(→ga nu naar vraag 12)***
- O 4 tot 6 maanden geleden ***(→ga nu naar vraag 12)***
- O 7 tot 12 maanden geleden ***(→ga nu naar vraag 12)***
- O meer dan 12 maanden geleden ***(→ga nu naar vraag 12)***

11. Wat zijn de voornaamste redenen om '10.000 Stappen' of een variant hiervan <u>niet</u> verder toe te passen? *(kruis **max. 2** bolletjes aan)*

- O we hebben hier nog niet concreet over nagedacht
- O onvoldoende steun van bestuur/diensthoofd
- O onvoldoende steun of motivatie onder de eigen collega's
- O onvoldoende (geplande) financiële ruimte
- O geen prioriteit/ niet geschikt voor onze lokale organisatie
- O verkiezen ander beweegproject
- O nood aan praktijkvoorbeelden en kennisondersteuning (bv. bijscholing)
- O Ander (*vul hieronder aan a.u.b.*):

………………………………………………………………………………………………………

→ Voor u <u>eindigt de vragenlijst hier</u>. Dank voor uw medewerking!

12. Wat heeft het meest bijgedragen tot de beslissing om '10.000 Stappen' of een variant hiervan verder toe te passen? *(kruis **max. 2** bolletjes aan)*

- O de bewijzen uit de wetenschappelijke studie van het pilootproject in Gent
- O de ervaringen van collega-organisaties uit een andere gemeente/regio
- O opdracht van het bestuur of diensthoofd
- O subsidies of andere ondersteuning
- O 10 000 Stappen is een gebruiksklaar product
- O Ander (*vul hieronder aan a.u.b.*):

………………………………………………………………………………………………………

→ Indien u 'Neen, maar we hebben wel de intentie' heeft geantwoord bij vraag 9
<u>eindigt de vragenlijst hier</u>. Dank voor uw medewerking!

13. Wat was/is de totale voorziene duur van de ’10.000 Stappen’-projecten of varianten hiervan die u in de periode mei 2009 tot april 2010 verder toegepast heeft? (*meerdere antwoorden mogelijk*).

- O 1 dag
- O 2 dagen tot 1 week
- O 2 weken tot 1 maand
- O 2 tot 3 maanden
- O 4 tot 6 maanden
- O 7 tot 12 maanden
- O Meer dan een jaar

14. Welk campagnebeeld is gebruikt binnen de periode mei 2009 tot heden voor de ondersteunde / uitgevoerde ‘10.000 stappen' projecten of varianten hiervan? *(beide keuzes mogelijk)*

- o Beeld van 10.000 stappen Vlaanderen
- o Eigen gecreëerd campagnebeeld
- o Ander (vul in):……………………………………………………..

→Indien u zonet (ook) ‘Beeld van 10.000 stappen Vlaanderen’ heeft aangeduid ga nu naar vraag 16

15. Waarom is het Vlaamse campagnebeeld van ’10.000 Stappen Vlaanderen’ niet gebruikt? Gelieve de reden hieronder te formuleren:

…………………………………………………………………………………………………….

→Ga nu naar vraag 17

16. Waarom is het Vlaamse campagnebeeld van ’10.000 Stappen Vlaanderen’ gebruikt? Gelieve de reden hieronder te formuleren:

…………………………………………………………………………………………………….

17. Hoeveel personeelsleden van de dienst hebben in de periode mei 2009 tot heden aan de '10.000 Stappen' projecten of varianten hiervan gewerkt?

Geef ook het aantal personeelsleden die niet aan de projecten meewerkten:

Aantal projectpersoneelsleden die meewerkten aan de projecten (*vul in* - bv. 3): ……….

Aantal personeelsleden die NIET meewerkten aan de projecten (*vul in* - bv. 1): ……….

18. Wanneer u binnen de periode mei 2009 tot april 2010 alle werkdagen van de betrokken personeelsleden in acht neemt:

hoeveel werkdagen (schatting) heeft uw dienst in het totaal aan de '10.000 Stappen'-projecten of varianten hiervan besteed binnen de periode mei 2009 tot april 2010?

(*vul in*): +-…………………werkdagen

19. Wat was/is voor uw dienst het totaal van de investering (excl. personeelskosten) in de '10.000 Stappen'- projecten of varianten hiervan in de periode mei 2009 tot april 2010?

(*vul in*): +-……………………euro

20. Op welke bewegingscontext(en) was/ is uw '10.000 Stappen'-campagne of variant hiervan in de periode april 2009 tot april 2010 gericht? *(kruis **1** of **meerdere** bolletjes aan)*

O beweging in de vrije tijd
O actieve verplaatsing (te voet, per fiets naar de winkel, werk, de post...)
O beweging op de werkvloer
O beweging thuis

21. Tot welke leeftijdsgroep(en) was/ is uw '10.000 Stappen'-campagne of variant hiervan in de periode april 2009 tot april 2010 gericht? *(kruis **1** of **meerdere** bolletjes aan)*

O kinderen en jongeren (<18 jaar)
O 18-tot-55-jarigen
O senioren (≥ 55 jaar)

22. Tot welke doelgroep(en) was/ is uw '10.000 Stappen'-campagne of variant hiervan in de periode april 2009 tot april 2010 gericht? *(kruis **1** of **meerdere** bolletjes aan)*

O volledige bevolking (al dan niet binnen bepaalde leeftijdscategorie van vorige vraag)

O het eigen personeel

O bedrijven

O scholen

O kansarmen

O Ander (*vul hieronder aan a.u.b.*):

……………………………………………………………………………………………………..

TOEPASSING - SPECIFIEK

23. Heeft u in de periode april 2009 tot april 2010 folders verspreid rond '10.000 Stappen' of een variant hiervan? (*kruis **1** bolletje aan*)

O Neen

O Ja, op éénmalige basis ***(→ga nu naar vraag 25)***

O Ja, deze werden meermaals verspreid of aangevuld ***(→ga nu naar vraag 25)***

24. Wat was/is de voornaamste reden om geen folders rond '10.000 Stappen' of een variant hiervan te verspreiden? *(kruis **max. 2** bolletjes aan)*

O We overwegen deze mogelijkheid nog

O Geen toestemming van overste of gemeentebestuur

O Te veel kosten

O Dit behoort niet tot onze kerntaken

O Nood aan meer inhoudelijke info/ondersteuning om deze folder op te stellen

O Geen tijd

O Ander *(vul hieronder aan a.u.b.*):

……………………………………………………………………………………………….

→ **Indien u zonet vraag 24 beantwoord heeft, ga dan nu naar vraag 26.**

25. Waar heeft u deze folders in de periode april 2009 tot april 2010 verspreid? *(kruis **1** of **meerdere** bolletjes aan)*

- O Bedrijven
- O Bibliotheek
- O Gemeentehuis/openbare diensten van de gemeente
- O Plaatselijk kantoor van (het) ziekenfonds(en)
- O Scholen
- O Lokaal dienstencentrum of OCMW
- O Ander *(vul hieronder aan a.u.b.)*:

……………………………………………………..

- O Lokale handelaars of warenhuis
- O Praktijk van huisartsen, kinesisten en/of osteopaten
- O Praktijk van diëtisten
- O Gemeentelijke/stedelijke sporthal
- O Apothekers
- O Seniorenclub
- O Wijkvereniging of -centrum

26. Heeft u in de periode april 2009 tot april 2010 gebruik gemaakt van baanaffiches* of signalisaties in het straatbeeld? (*kruis **1** bolletje aan*)

- O Neen
- O Ja, op éénmalige basis ***(→ga nu naar vraag 28)***
- O Ja, deze werden meermaals verspreid of aangevuld ***(→ga nu naar vraag 28)***

(*)*baanaffiche:* affiche voor op straat, langs wegen, bij parkings

27. Wat was/is de voornaamste reden om in de periode april 2009 tot april 2010 geen baanaffiches of signalisaties te gebruiken? *(kruis **max. 2** bolletjes aan)*

- O We overwegen deze mogelijkheid nog
- O Wetgeving bemoeilijkte dit
- O Geen toestemming van overste of gemeentebestuur
- O Nood aan meer inhoudelijke info/ondersteuning om deze baanaffiches of signalisatie op te maken
- O Te veel kosten
- O Dit behoort niet tot onze kerntaken
- O Geen tijd
- O Ander *(vul hieronder aan a.u.b.)*:

…………………………………………………………………………………………………….

→ **Indien u zonet vraag 27 beantwoord heeft, ga dan nu naar vraag 29.**

28. Waar heeft u in de periode april 2009 tot april 2010 deze baanaffiches of signalisaties opgehangen/geplaatst? *(kruis **1 of meer** bolletjes aan)*

O Bedrijven

O Parking van gemeentelijk / stedelijk centrum

O In lokale straten (gemeentelijk / stedelijk centrum)

O In lokaal park

O Langs gemeentelijke / stedelijke autowegen

O Langs recreatieve wandelroutes

O Langs recreatieve fietsroutes

O Ander *(vul hieronder aan a.u.b.)*:

…………………………………………………………………………………………….

29. Heeft u in de periode april 2009 tot april 2010 gebruik gemaakt van gewone posters? (*kruis **1** bolletje aan*)

O Neen

O Ja, op éénmalige basis ***(→ga nu naar vraag 31)***

O Ja, deze werden meermaals verspreid of aangevuld ***(→ga nu naar vraag 31)***

30. Wat was/ is de voornaamste reden om in de periode april 2009 tot april 2010 geen gewone posters te verspreiden?

O We overwegen deze mogelijkheid nog

O Geen toestemming van overste of gemeentebestuur

O Nood aan meer inhoudelijke info/ondersteuning om deze poster op te maken

O Te veel kosten

O Dit behoort niet tot onze kerntaken

O Geen tijd

O Ander *(vul hieronder aan a.u.b.)*:

…………………………………………………………………………………………….

→ **Indien u zonet vraag 30 beantwoord heeft ga dan nu naar vraag 32**

31. Waar heeft u in de periode april 2009 tot april 2010 deze gewone posters verspreid?

O Bedrijven
O Bibliotheek
O Gemeentehuis/openbare diensten van de gemeente
O Plaatselijke kantoren van het ziekenfonds
O Scholen
O Lokaal dienstencentrum of OCMW
O Lokale handelaars of warenhuis
O Praktijk van huisartsen, kinesisten en/of osteopaten
O Praktijk van diëtisten
O Apothekers
O Gemeentelijke/stedelijke sporthal
O Seniorenclub
O Wijkvereniging of –centrum
O Ander *(vul hieronder aan a.u.b.)*:

……………………………………………………………………………………………….

32. Heeft u in de periode april 2009 tot april 2010 gebruik gemaakt van interne of externe mediakanalen* om uw '10.000 Stappen'-campagne of een variant hiervan te promoten? (*kruis **1** bolletje aan*)

O Neen
O Ja, op éénmalige basis ***(→ga nu naar vraag 34)***
O Ja, deze werden meermaals gebruikt ***(→ga nu naar vraag 34)***

(*)Mediakanalen: ledenbladen, kranten voor de inwoners (lokaal, regionaal, of nationaal), al dan niet streekgebonden tijdschriften en magazines, regionale of nationale televisie

33. Wat was/is de voornaamste reden om geen mediakanalen te gebruiken? *(kruis **max. 2** bolletjes aan)*

O We overwegen deze mogelijkheid nog
O Geen toestemming van overste of gemeentebestuur
O Dit behoort niet tot onze kerntaken
O Te veel kosten
O Geen tijd
O Ander *(vul hieronder aan a.u.b.)*:

……………………………………………………………………………………………….

→ **Indien u zonet vraag 33 beantwoord heeft, ga dan nu naar vraag 35.**

34. Welke mediakanalen werden in de periode mei 2009 tot april 2010 aangewend? *(kruis **1** of **meerdere** bolletjes aan)*

- O Ledenblad
- O Gemeentelijk/stedelijk infoblad
- O De Streekkrant
- O Regionaal tijdschrift of magazine
- O Nationale krant (bv. Het Nieuwsblad)
- O Websites en/of e-zines
- O Vakspecifiek tijdschrift medische sector
- O Regionale televisie
- O Radio
- O Persconferentie/ persbericht
- O Ander *(vul hieronder aan a.u.b.)*:

………………………………………………

35. Heeft u in de periode april 2009 tot april 2010 pedometers of stappentellers ingeschakeld in functie van uw '10.000 Stappen'-campagne of variant hiervan? (*kruis **1** bolletje aan*)

- O Neen
- O Ja ***(→ga naar vraag 37)***

36. Wat was/is de voornaamste reden om geen pedometers of stappentellers in te schakelen? *(kruis **max. 2** bolletjes aan)*

- O We overwegen deze mogelijkheid nog
- O Geen toestemming van overste of gemeentebestuur
- O Te veel kosten
- O Dit behoort niet tot onze kerntaken
- O Nood aan meer inhoudelijke info/ondersteuning om acties rond stappentellers op te zetten
- O Geen tijd
- O Ander: *(vul hieronder aan a.u.b.)*:

…………………………………………………………………………………………….

→ **Indien u zonet vraag 36 beantwoord heeft, ga dan nu naar vraag 41.**

37. Welk merk hebben de pedometers of stappentellers die zijn ingeschakeld/aangekocht in de periode april 2009 tot april 2010? *(kruis **1** of **meerdere** bolletjes aan)*

O Yamax Digiwalker

O Omron - type 'Vital Steps' (HJ-005-E)

O Omron - type 'Active Steps' (HJ-109-E)

O Omron - type 'Walking Style I' (HJ-152-E)

O Omron - type 'Walking Style II' (HJ-113-E)

O Ander *(vul hieronder aan a.u.b.)*:

…………………………………………………………………………………………………….

38. Hoe werden/worden de pedometers of stappentellers in de periode april 2009 tot april 2010 gebruikt/aangeboden? *(kruis **1** of **meerdere** bolletjes aan)*

O gratis aanbod (gratis verdeling)

O voor individuele verkoop

O voor individuele verhuur tegen betaling

O voor individuele verhuur en gratis (al dan niet met waarborg)

O voor groepsverhuur (bv. werking met verhuurkoffers)

O Ander *(vul hieronder aan a.u.b.)*:

……………………………………………………………………………………………………

39. Werd/wordt er steeds een begeleidend (stappen)boekje* aan de koper/huurder aangeboden bij de pedometer of stappenteller? (*kruis **1** bolletje aan*)

**nooit**	**zelden**	**soms**	**vaak**	**altijd**
O	O	O	O	O

40. Waar werden/worden in de periode april 2009 tot april 2010 de pedometers of stappentellers aangeboden? *(kruis **1** of **meerdere** bolletjes aan)*

O Bedrijven
O In het gemeente-/stadshuis of openbare diensten van de gemeente/stad
O Lokale dienstencentrum of OCMW
O Plaatselijk kantoor / verkooppunt van (het) ziekenfonds(en)
O Scholen
O Bibliotheek
O Praktijk van huisartsen, kinesisten en/of osteopaten
O Apothekers
O Seniorenclub
O Wijkvereniging of –centrum
O Ziekenhuis
O Ander *(vul hieronder aan a.u.b.)*:

……………………………………………………..

41. Heeft u in de periode april 2009 tot april 2010 samengewerkt met andere (gemeentelijke/stedelijke) diensten, instanties of verenigingen in functie van uw '10.000 Stappen'-campagne of variant hiervan? (*kruis **1** bolletje aan*)

O Neen
O Ja ***(→ga nu naar vraag 43)***

42. Wat is de voornaamste reden voor het feit dat er niet is samengewerkt met andere (gemeentelijke/stedelijke) diensten, instanties of verenigingen in de periode april 2009 tot april 2010? *(kruis **max. 2** bolletjes aan)*

O We overwegen deze mogelijkheid nog
O De andere instantie(s) had(den)/heeft (hebben) geen interesse of de relatie is niet positief
O Geen toestemming van overste of gemeentebestuur
O Dit bood geen meerwaarde aan de campagne
O Dit behoort niet tot onze kerntaken
O Nood aan kennisondersteuning voor het opzetten van partnerships
O Geen tijd
O Ander *(vul hieronder aan a.u.b.)*:

……………………………………………………………………………………………………

→ **Indien u zonet vraag 42 beantwoord heeft, ga dan nu naar vraag 45.**

43. Met welke instanties/verenigingen heeft u in de periode april 2009 tot april 2010 samengewerkt in functie van het bereiken van de beoogde doelgroep(en)? *(kruis **1** of **meerdere** bolletjes aan)*

- O Preventieadviseur, bedrijfsleider of medische dienst van bedrijf
- O Bibliotheken
- O Gemeente-/stadsbestuur
- O Gemeentelijke/stedelijke sportdienst
- O Gemeentelijke/stedelijke dienst mobiliteit
- O Gemeentelijke/stedelijke dienst Welzijn of Gezondheid
- O Lokaal dienstencentrum of OCMW
- O Provinciale dienst (bv. sport, gezondheid, mobiliteit,…)
- O Huisartsen, kinesisten en/of osteopaten
- O OKRA of S-Sport
- O Ziekenfonds(en)
- O Apothekers
- O Lokaal Gezondheidsoverleg (LOGO)
- O Scholen
- O Seniorenclub
- O Wijkvereniging of -centrum
- O Ander: *(vul hieronder aan a.u.b.)*:

…………………………………………………..

44. Is er in in de periode april 2009 tot april 2010 in functie van uw '10.000 Stappen'-campagne of variant hiervan een werkgroep gevormd met leden van de samenwerkende instanties/ verenigingen? (*kruis **1** bolletje aan*)

- O Neen,
- O Ja, maar zonder het Lokaal gezondheidsoverleg
- O Ja, en met het Lokaal gezondheidsoverleg

45. Heeft u in de periode april 2009 tot april 2010 in functie van uw '10.000 Stappen'-campagne of variant hiervan de beoogde doelgroepen op een persoonlijke manier gecontacteerd of laten contacteren (= per mail, per PERSOONLIJKE brief*, of telefonisch)? (*kruis **1** bolletje aan*)

- O Neen
- O Ja, op éénmalige basis ***(→ga nu naar vraag 47)***
- O Ja, deze werden meermaals verspreid of aangevuld ***(→ga nu naar vraag 47)***

46. Wat was/is de voornaamste reden voor het feit dat u de beoogde doelgroepen niet op een persoonlijke manier gecontacteerd of laten contacteren heeft? *(kruis **max. 2** bolletjes aan)*

- O We overwegen deze mogelijkheid nog
- O Geen toestemming van overste of gemeentebestuur
- O Te veel kosten
- O Dit behoort niet tot onze kerntaken
- O Geen tijd
- O Ander *(vul hieronder aan a.u.b.)*:

……………………………………………………………………………………………………

→ **Indien u zonet vraag 46 beantwoord heeft, ga dan nu naar vraag 48**

47. Gelieve voor de periode april 2009 tot april 2010 bondig het aantal personen te noteren dat u op een persoonlijk manier heeft gecontacteerd?

Aantal (bv. 'helft van alle leden', '500 personen op infobeurs',...):

(*vul in*): ……

48. Welke andere concrete acties werden in de periode april 2009 tot april 2010 georganiseerd specifiek in functie van de '10.000 Stappen'-campagne of variant hiervan?

Geef bij uw keuze ook aan HOEVEEL KEER u deze actie ondernomen heeft georganiseerd.

- O Interne acties voor het eigen personeel (bv. stappenwedstrijd, ...):
  (vul in) : ………. keer
- O Infostand of promo-acties rond ‘10.000 Stappen’ gedurende publieke evenementen:
  (vul in) : ………. keer
- O Aangeven van afstanden tussen publieke plaatsen in aantal stappen (bv. van gemeente-/stadhuis tot station, …):
  (vul in) : ………. keer
- O Uitstippelen van publieke (recreatieve) wandelparcours in aantal stappen:
  (vul in) : ………. keer
- O Themawandelingen in de gemeente / stad met of zonder stappentellers (bv. bedrijfsincentives; natuurgebonden, toeristische, of culinaire themawandelingen,…):
  (vul in): ………. keer
- O Geen andere concrete acties
- O Anders *(vul in)*:……………………………………………………………………………..
  (vul in): ………. keer

49. Heeft u in de periode april 2009 tot april 2010 nog andere zaken toegepast in uw '10.000 Stappen'-campagne die tot nu toe nog niet aan bod zijn gekomen? (bv. banners in plaats van signalisaties, sms-berichten in plaats van emails, etc.) Indien ja, gelieve dit te formuleren a.u.b.

Ter info: volgende items zijn reeds bevraagd: folders, baanaffiches en signalisaties in het straatbeeld, posters, media, pedometers, persoonlijk contact met de doelgroep, en concrete acties.

O Neen

O Ja (*vul in*):

……

……

……

50. Heeft u in de periode april 2009 tot april 2010 de website van '10.000 Stappen', met name www.10000stappen.be al gebruikt?

O Neen

O Ja, al gebruikt maar niet gepromoot

O Ja, al gebruikt en ook gepromoot bij onze doelgroep

DEEL 2 – VERANKERING 10.000 STAPPEN

1a. Werden de doelstellingen van uw '10.000 stappen'-project of variant op papier gezet in de periode mei 2009 tot april 2010?

Ja — Neen — Niet zeker / niet van toepassing

↓ (Niet zeker / niet van toepassing) *Ga nu naar vraag 2*

↓ (Ja) **1b**. Indien ja, sinds hoeveel jaren werden die geschreven doelstellingen toen al nagestreefd binnen uw '10.000 stappen'-project?

jaar / jaren (*vul aantal in)*

↓ (Ja, Neen)

1c. Van alle projectcomponenten van uw '10.000 stappen'-project of variant die een doelstelling op papier hadden kunnen krijgen, voor welk deel hiervan werd er werkelijk ook een geschreven doelstelling voorzien in de periode mei 2009 tot april 2010 (ruwe schatting)?

Geen<br>1	Klein deel<br>2	Groot deel<br>3	Allemaal<br>4

2a. Werden de plannen of procedures van projectuitvoering van uw '10.000 stappen'-project of variant op papier gezet in de periode mei 2009 tot april 2010?

Ja — Neen — Niet zeker / niet van toepassing

↓ (Niet zeker / niet van toepassing) *Ga nu naar vraag 3*

↓ (Ja) **2b**. Indien ja, sinds hoeveel jaren werden deze uitgeschreven plannen of procedures van projectuitvoering toen al nagestreefd binnen uw '10.000 stappen'-project?

jaar / jaren (*vul aantal in)*

↓ (Ja, Neen)

2c. Van alle projectcomponenten van uw '10.000 stappen'-project of variant die een plan van uitvoering hadden kunnen krijgen, voor welk deel hiervan werd er ook werkelijk een geschreven plan voorzien in de periode mei 2009 tot april 2010 (ruwe schatting)?

Geen<br>1	Klein deel<br>2	Groot deel<br>3	Allemaal<br>4

3a. Werd er een tijdsschema voor de uitvoering van activiteiten van uw '10.000 stappen'-project of variant op papier gezet in de periode mei 2009 tot april 2010?

Ja — Neen — Niet zeker / niet van toepassing

↓ (Ja)

3b. Indien ja, sinds hoeveel jaren werd een uitgeschreven tijdschema toen al nagestreefd binnen uw '10.000 stappen'-project?

jaar / jaren (*vul aantal in*)

↓ (Niet zeker / niet van toepassing)

Ga nu naar vraag 4

↓

3c. Van alle projectcomponenten van uw '10.000 stappen'-project of variant die een tijdschema hadden kunnen krijgen, voor welk deel hiervan werd er ook werkelijk een geschreven plan van uitvoering voorzien in de periode mei 2009 tot april 2010 (ruwe schatting)?

Geen<br>1	Klein deel<br>2	Groot deel<br>3	Allemaal<br>4

4a. Werden de strategieën voor de uitvoering van het '10.000 stappen'-project of variant aangepast om in te spelen op lokale omstandigheden in de periode mei 2009 tot april 2010?

Ja — Neen — Niet zeker / niet van toepassing

↓ (Ja)

4b. Indien ja, sinds hoeveel jaren werden deze aangepaste strategieën toen al nagestreefd binnen uw '10.000 stappen'-project?

jaar / jaren (*vul aantal in*)

↓ (Niet zeker / niet van toepassing)

Ga nu naar vraag 5

↓

4c. Van alle projectcomponenten van uw '10.000 stappen'-project of variant die hadden kunnen aangepast worden aan lokale omstandigheden, welk deel hiervan werd ook werkelijk aangepast in de periode mei 2009 tot april 2010 (ruwe schatting)?

Geen<br>1	Klein deel<br>2	Groot deel<br>3	Allemaal<br>4

5a. Werd er een formele evaluatie van uw '10.000 stappen'-project of variant uitgevoerd in de periode mei 2009 tot april 2010?

Ja | Neen | Niet zeker / niet van toepassing

↓ (Ja)

5b. Indien ja, hoeveel jaren werd uw '10.000 stappen'-project toen al geëvalueerd?

jaar / jaren (*vul aantal in*)

↓ (Niet zeker / niet van toepassing)

Ga nu naar vraag 6

↓

5c. Van alle projectcomponenten van uw '10.000 stappen'-project of variant die hadden kunnen geëvalueerd worden, welk deel hiervan werd ook werkelijk geëvalueerd (ruwe schatting) in de periode mei 2009 tot april 2010?

Geen<br>1	Klein deel<br>2	Groot deel<br>3	Allemaal<br>4

BESTUURLIJK

6a. Werd er een supervisor (bv. diensthoofd) aangewezen die toezicht hield op uw '10.000 stappen'-project of variant in de periode mei 2009 tot april 2010?

Ja | Neen | Niet zeker / niet van toepassing

↓ (Ja)

6b. Indien ja, hoeveel jaren werd er toen al door een supervisor toegezien op uw '10.000 stappen'-project?

jaar / jaren (*vul aantal in*)

↓ (Niet zeker / niet van toepassing)

Ga nu naar vraag 7

↓

6c. Van alle projectcomponenten van uw '10.000 stappen'-project of variant die supervisie hadden kunnen krijgen, welk deel hiervan heeft werkelijk ook supervisie gekregen in de periode mei 2009 tot april 2010 (ruwe schatting)?

Geen<br>1	Klein deel<br>2	Groot deel<br>3	Allemaal<br>4

7a. Werden er taakomschrijvingen op papier gezet voor personeel betrokken bij uw '10.000 stappen'-project of variant in de periode mei 2009 tot april 2010?

Ja | Neen | Niet zeker / niet van toepassing

↓ (Ja)

7b. Indien ja, sinds hoeveel jaren werd deze taakomschrijving toen al door het betrokken personeel nagestreefd binnen uw '10.000 stappen'-project ?

jaar / jaren (*vul aantal in)*

↓ (Niet zeker / niet van toepassing)

Ga nu naar vraag 8

7c. Hoeveel personeelsleden die betrokken waren bij uw '10.000 stappen'-project of variant hadden een geschreven taakomschrijving in de periode mei 2009 tot april 2010 (ruwe schatting)?

Geen	Weinig	De meeste	Allemaal
1	2	3	4

8a. Werden evaluaties van uw '10.000 stappen'-project of variant op een zelfde manier uigevoerd als evaluaties van de meeste andere projecten van uw organisatie in de periode mei 2009 tot april 2010?

Ja | Neen | Niet zeker / niet van toepassing

↓ (Ja)

8b. Indien ja, hoeveel jaren werden deze evaluaties van uw '10.000 stappen'-project op eenzelfde manier uigevoerd als evaluaties van de andere projecten

jaar / jaren (*vul aantal in)*

↓ (Niet zeker / niet van toepassing)

Ga nu naar vraag 9

8c. Welk deel van de evaluatie(s) van uw '10.000 stappen'-project of variant werd op een zelfde manier uitgevoerd als evaluaties van de meeste andere projecten van uw organisatie in de periode mei 2009 tot april 2010 (ruwe schatting)?

Geen	Klein deel	Groot deel	Allemaal
1	2	3	4

BEHOUD

9a. Werd er een vast personeelslid toegewezen aan de uitvoering van uw '10.000 stappen'-project of variant in de periode mei 2009 tot april 2010?

Ja | Neen | Niet zeker / niet van toepassing

↓ (Ja)

9b. Indien ja, hoeveel jaren werd er toen al een vast personeelslid toegewezen aan de uitvoering van uw '10.000 stappen'-project?

jaar / jaren (*vul aantal in)*

↓ (Niet zeker / niet van toepassing)

Ga nu naar vraag 10

↓

9c. Welk deel van de vaste personeelsleden was betrokken bij de uitvoering van uw '10.000 stappen'-project of variant in de periode mei 2009 tot april 2010 (ruwe schatting)?

Geen	Klein deel	Groot deel	Allemaal
1	2	3	4

10a. Werd er administratief personeel van uw organisatie betrokken bij het pleiten voor het verder zetten van uw '10.000 stappen'-project of variant in de periode mei 2009 tot april 2010?

Ja | Neen | Niet zeker / niet van toepassing

↓ (Ja)

10b. Indien ja, hoeveel jaren werd er toen al administratief personeel betrokken bij het pleiten voor het verder zetten van uw '10.000 stappen'-project?

jaar / jaren (*vul aantal in)*

↓ (Niet zeker / niet van toepassing)

Ga nu naar vraag 11

↓

10c. Hoe actief was dit administratief personeel betrokken bij het pleiten voor het verder zetten van uw '10.000 stappen'-project of variant in de periode mei 2009 tot april 2010?

Helemaal niet	Weinig	Matig	Heel actief
1	2	3	4

11a. Werden er nog andere personeelsleden, naast deze die het project uitvoerden, actief betrokken bij uw '10.000 stappen'-project of variant in de periode mei 2009 tot april 2010?

Ja | Neen | Niet zeker / niet van toepassing

↓ (Ja)

11b. Indien ja, hoeveel jaren hadden deze andere personeelsleden toen al bijgedragen aan uw '10.000 stappen'-project?

jaar / jaren (*vul aantal in)*

↓ (Niet zeker / niet van toepassing)

Ga nu naar vraag 12

↓

11c. Van alle personeelsleden van uw organisatie, welk deel heeft er werkelijk bijgedragen tot uw '10.000 stappen'-project of variant in de periode mei 2009 tot april 2010?

Geen<br>1	Klein deel<br>2	Groot deel<br>3	Allemaal<br>4

ONDERSTEUNING

12a. Werd de status van uw '10.000 stappen'-project of variant in de periode mei 2009-april 2010 al veranderd van pilootproject (niet-permanent) naar permanent project binnen uw organisatie?

Ja | Neen | Niet zeker / niet van toepassing

↓ (Ja)

12b. Indien ja, hoeveel jaren had uw '10.000 stappen'-project toen al een status van permanent project?

jaar / jaren (*vul aantal in)*

↓ (Niet zeker / niet van toepassing)

Ga nu naar vraag 13

↓

12c. In welke mate had uw '10.000 stappen'-project of variant in de periode mei 2009-april 2010 een status van permanent project binnen uw organisatie?

Helemaal niet<br>1	Weinig<br>2	Matig<br>3	Volledig<br>4

13a. Werd er binnen uw organisatie aan uw '10.000 stappen'-project of variant permanente materiële ondersteuning gegeven in de periode mei 2009-april 2010?

Ja | Neen | Niet zeker / niet van toepassing

↓ (Ja)

13b. Indien ja, sinds hoeveel jaren had uw '10.000 stappen'-project toen al materiële ondersteuning gekregen?

jaar / jaren (*vul aantal in)*

↓ (Niet zeker / niet van toepassing)

Ga nu naar vraag 14

↓ (Ja, Neen)

13c. Van alle materiële ondersteuning die uw '10.000 stappen'-project of variant nodig had, welk deel hiervan heeft het werkelijk ook gekregen in de periode mei 2009 tot april 2010 (ruwe schatting)?

Geen	Klein deel	Groot deel	Alles wat het nodig had
1	2	3	4

14a. Waren de financieringbronnen van uw '10.000 stappen'-project of variant gelijkaardig als die van andere gevestigde projecten binnen uw organisatie in de periode mei 2009-april 2010?

Ja | Neen | Niet zeker / niet van toepassing

↓ (Ja)

14b. Indien ja, sinds hoeveel jaren waren financieringbronnen van uw '10.000 stappen'-project toen al gelijkaardig als die van andere gevestigde projecten binnen uw organisatie?

jaar / jaren (*vul aantal in)*

↓ (Niet zeker / niet van toepassing)

Ga nu naar vraag 15

↓ (Ja, Neen)

14c. Hoe permanent waren deze financieringsbronnen van uw '10.000 stappen'-project of variant in de periode mei 2009-april 2010?

Helemaal niet	Weinig	Matig	Volledig permanent
1	2	3	4

15a. Werd het personeel dat het meest betrokken was bij uw '10.000 stappen'-project of variant betaald met middelen uit een stabiele financieringsbron in de periode mei 2009-april 2010?

Ja — Neen — Niet zeker / niet van toepassing

Ja ↓

15b. Indien ja, sinds hoeveel jaren werd het personeel dat het meest betrokken was bij het project toen al betaald met middelen uit een stabiele financieringsbron?

jaar / jaren (*vul aantal in)*

↓

Neen ↓ (naar 15c)

Niet zeker / niet van toepassing ↓

Einde - bedankt voor uw tijd!

15c. Hoe permanent was deze financiering van personeel dat het meest betrokken was bij uw '10.000 stappen'-project of variant in de periode mei 2009-april 2010?

Helemaal niet<br>1	Weinig<br>2	Matig<br>3	Volledig permanent<br>4

Einde - bedankt voor uw tijd!
